# Patient and public involvement in numerical aspects of trials (PoINT): exploring patient and public partners experiences and identifying stakeholder priorities

**DOI:** 10.1186/s13063-021-05451-x

**Published:** 2021-07-28

**Authors:** Beatriz Goulao, Hanne Bruhn, Marion Campbell, Craig Ramsay, Katie Gillies

**Affiliations:** Health Services Research Unit, University of Aberdeen, Aberdeen, UK

## Abstract

**Background and aims:**

Patient and public involvement is increasingly common in trials, but its quality remains variable in a lot of settings. Many key decisions in trials involve numbers, but patients are rarely involved in those discussions. We aimed to understand patient and public partners’ experiences and opinions regarding their involvement in numerical aspects of research and discuss and identify priorities, according to multiple stakeholders, around the most important numerical aspects in trials to involve patients and the public in.

**Methods:**

The study had two stages: (1) online focus groups with patient and public partners recruited via online platforms and analysed using inductive thematic analysis and (2) online priority setting meeting with UK- and Ireland-based stakeholders and following James Lind Alliance methodology. Pre-selected numerical aspects were introduced prior to the meeting and discussed and prioritised based on a voting system.

**Results:**

In stage 1, we held two focus groups with patient and public partners (n = 9). We identified four themes in the analysis: “Determinants of PPI in numerical aspects”, “Identity and roles”, “Impact of involving patients and the public in numerical aspects”. Patient and public partners believed being involved in numerical aspects of research is important and should be facilitated, but communication about these aspects needs to be clearer. An environment and relationship with researchers that facilitates that will include time for discussion, support to improve knowledge and confidence, clear language and definitions and trust. Patient and public partners perceive their role as bringing an outsider perspective and were mainly interested in involvement in assumptions and dissemination of quantitative research. They believed this can lead to more transparency and improve their experience by making involvement more meaningful.

In stage 2, we identified twelve numerical aspects of trials to be prioritised. We held a priority setting meeting with 14 stakeholders, which led to the selection of three priority numerical aspects in patient and public involvement: target differences, interpretation of results and cost-effectiveness. Participants felt all aspects should be considered for involvement and their communication needs to ensure a shared level of understanding to avoid power imbalances.

**Conclusions:**

Our work shows the importance of involving patient and public partners in numerical aspects of trials by assessing their experiences and motivations for the first time and discussing and prioritising which numerical aspects of trials are the most important for patients and the public to contribute to. Our research provides a platform for future efforts to improve patient and public involvement in trials and a prioritised set of future research foci.

## Background

Involvement of patients and/or public in research (herein referred to as Patient and Public Involvement (PPI)) is about carrying out research with or by members of the public, including patients (http://www.invo.org.uk/). PPI activity has become increasingly common for three main reasons: it makes research better, for example by improving recruitment to trials [1]; its supporters believe it is the right thing to do, particularly when research is publicly funded; it is a requirement from major funders, including the National Institute for Health Research (NIHR) in the UK [2].

In a trial context, patient and public partners have to date been primarily involved in steering committees, ethical review, and protocol development [3], but involvement remains variable in many settings [4, 5]. One particular area which has received little attention in trials is involvement in its numerical aspects, even though there is increased interest in involving patients in methodological aspects of trial design [6]. Trials are heavily quantitatively focussed and key decisions are based on numerical aspects (for example, what the target difference or the randomisation ratio should be etc.). Improving involvement and communication related to these aspects could help develop strong and productive relationships, the number one methodological priority for patient and public involvement in clinical trials [7]. Statisticians and health economists have raised the importance of patient and public involvement in the design and analysis of quantitative research with perceived benefits including more robust data interpretation and research [8, 9]. We recently conducted a survey of UK-based trialists and found the extent to which patients and the public are involved in numerical discussions varies considerably and that trialists find the communication of numerical and statistical aspects challenging, but important. Key barriers to improve patient and public involvement in numerical aspects of research included a perceived lack of interest from patient and public partners as well as a lack of understanding about how and when this type of involvement should happen [10].

To our knowledge, there has been no previous research to understand patient’s and the public’s perspectives and interest in involvement in numerical aspects of trials. If there is interest, then what numerical aspects should we be looking at? Our work sought to address that gap and was divided into two stages: stage 1, understand patient and public partners’ experiences and opinions regarding their involvement in numerical aspects of research and trials, and, stage 2, discuss and identify priorities, according to multiple stakeholders, around the most important numerical aspects in trials to involve patients and the public in.

## Methods

### Stage 1: Focus groups with patient and public partners

To gain an understanding of patient and public opinions and perspectives of involvement with numerical aspects of research and trials, two focus groups were conducted.

Eligible participants were adults, UK-based patient and public partners (defined as patients and members of the public with previous experience of being involved in research). Participants were recruited via two main channels: open adverts (via email, Twitter and People in Research) and targeted invitation through existing PPI groups. We aimed to recruit a maximum of five participants per focus group following recent guidelines on conducting online group discussions [11].

Focus groups were facilitated by a member of the research team (BG) who was assisted by a colleague (HB or Rumana Newlands) at each focus group. The semi-structured topic guide covered the following: experiences of PPI (for example, what stages of research they had been involved in), experiences of involvement in numerical aspects of research, barriers and facilitators to involvement in numerical aspects of research and consequences of involvement in numerical aspects of research. The discussion was kept open in terms of type of research (any type of quantitative research was relevant, not just trials). However, to exemplify opportunities for involvement in numerical aspects, we used trials as a case study. Specifically, we used a diagram discussing different stages in trials (from trial design to its dissemination) and an example of a numerical aspect, non-inferiority margins, to aid discussion.

Participants taking part in the focus group were invited to submit basic demographic data (gender, age category, ethnicity, and previous involvement experience). The focus groups were held remotely on Zoom and audio-recorded and lasted 2 h. The audio-recordings were transcribed verbatim*,* checked for accuracy and identifiable information redacted.

The data were analysed using an iterative thematic approach [12]. The first full transcript was coded inductively by one researcher (BG) and a preliminary thematic framework was developed. A second researcher (HB) applied the framework to the first full transcript. A discussion about disagreements and fit of the thematic framework and its appropriateness was held until agreement was reached and the agreed framework was reapplied. Word and Excel were used to conduct the thematic analysis.

### Stage 2: Prioritisation exercise

#### 2A) Numerical aspects to prioritise

To identify the numerical aspects of trials to include in the priority setting meeting, we conducted a systematic review of methods to elicit patient and public’s opinions on numerical aspects of research. The review’s details will be published separately, but the review’s protocol is available upon request. In summary, potentially relevant titles and abstracts were screened by one reviewer (BG), with any uncertainties discussed with a second reviewer (MC, KG, or CR). Six randomly selected abstracts (around 1% of the total number of abstracts retrieved) were reviewed independently by a second reviewer (MC, KG, or CR) for quality assurance. Full-text articles were obtained for the titles and abstracts identified as potentially relevant. These were provisionally categorised according to method of elicitation of patient/public views (if detailed in the abstract). A reviewer (BG) screened the full-text articles and extracted information, after having screened and extracted information from a practice sample of articles. Where there was uncertainty regarding whether or not a study should be included for data extraction, the opinion of a second reviewer (MC, KG, CR) was sought, and the study discussed until consensus was reached. Data were extracted verbatim on the methodological details by one author (BG), including type of article, year of publication, field, aim, participants involved, method used for elicitation of numerical aspects, numerical aspects being elicited and reasons to involve non-experts.

The review was additionally supplemented with information collected in a previous survey of UK trialists conducted by our group [10]. Specifically, the question from the survey about current practice in terms of involving patients and the public in numerical aspects of trials was used to supplement the literature review list. The survey included 187 respondents and collected data from June to July 2019.

Finally, the full list of all identified numerical aspects extracted was collated and discussed as a group, which included the study core team and collaborators: a statistician (BG), trial methodologist (KG), chief investigators (CR, MC), a patient partner (Richard Caie) and a health economist (Dwayne Boyers). At this point, three new aspects were added to the list.

#### 2B) Priority setting meeting

The priority setting meeting was a half day event, held remotely, of plenary and small group discussion, chaired by a James Lind Alliance (JLA) Senior Adviser. We brought together representatives from key stakeholder groups to determine the top 3 list of priorities from the numerical aspects identified in the previous stage. We followed JLA guidelines to conduct online group discussions and aimed to have a maximum of 15 participants since it would allow us to hold three smaller group discussion with 5 participants each [11].

The priority setting methodology was an adaptation of the standard approach described in the JLA Guidebook, namely using small and whole group discussions in a meeting with a particular emphasis on the top 3 [13]. Adaptions were related to the online nature of the meeting, as well as the fact that we had fewer aspects to prioritise than a traditional JLA priority setting exercise. The session was half day event instead of a full day to avoid screen fatigue; for that reason and given the number of aspects discussed, we compacted breakout sessions as described in the next paragraph.

All attendees were provided with the list of numerical aspects in advance of the meeting, as well as lay explanations of what each aspect meant, to allow time to familiarise themselves with the aspects and consider their thoughts on the importance of each one. A presentation about the meeting’s aims and other relevant documents, including participants’ introductions, were sent ahead of the meeting. A JLA facilitator along with two facilitators (BG, HB) led each of three small groups, which consisted of even representation of the stakeholder groups. The facilitators acted as neutral guides for the process and ensured equal participation to minimise authority effects. After an introductory plenary session with the entire group, the three small groups were convened and asked to discuss and prioritise three top aspects. These initial small groups were then mixed for the second round of discussion and a second prioritisation to ensure exposure to a range of ideas and eliminate the potential bias of group think. The exception was patient partners, who were allocated the same moderator (who did not participate in the discussion) in both rounds of discussion. Finally, the small groups all came back together in a plenary session to vote on the final prioritised list. Participants were invited to select two aspects that they felt should be prioritised in terms of patient and public involvement in numerical aspects of trials by voting anonymously via SurveyMonkey [14]. The three items with the highest percentage of votes were selected as the top 3. Participants were asked to reflect on the selection. Stata 16 [15] was used to summarise the priority setting results.

## Results

### Stage 1: Focus group with patient and public partners

We conducted two focus groups in December 2020 with a total of nine participants. One of the nine participants did not submit their basic demographic data. Most of the participants were older than 55 and the majority were white. All participants had previous experience of being involved in research as a patient or public partner, but the range of experiences varied from more experienced patient and public partners that had been involved in the design of projects and in applying for funding to patient and public partners that had just started their involvement journey and had limited knowledge of the research lifecycle. More details are available in Table 1.

**Table 1:** Focus group participant characteristics (stage 1)

Characteristics	Frequency (total = 9 participants)
Gender	
Female	4
Male	3
Missing or prefer not to say	2
Age category	
18–35	1
36–55	0
> 55	7
Missing	1
Ethnicity	
White	7
Missing	2
Previous experience of being a patient and public partner in research	
Yes	9
No	0

**Fig. 1:**
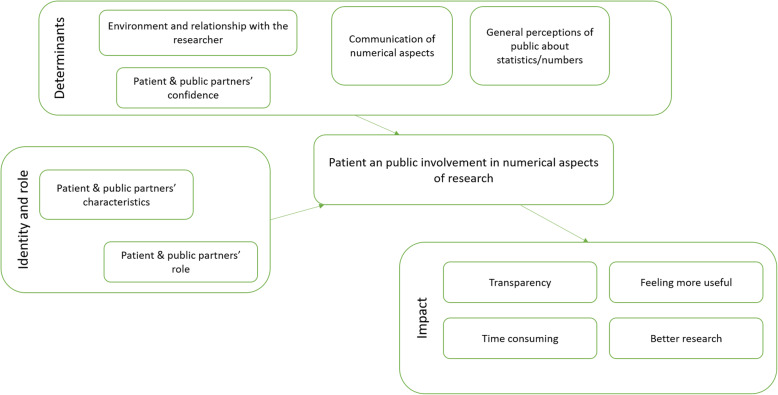
Visual representation of the thematic analysis results (stage 1)

We identified three main themes in our analysis: (1) determinants of patient and public involvement in numerical aspects of research, (2) identity and role and (3) impact of involving patients and the public in numerical aspects. Each theme was then divided into sub-themes and they are described below. The themes identified in our analysis are presented in Fig. 1. Table 2 summarises sub-themes and includes illustrating quotes. Each quote identifies the participant (P) and the focus group (FG) using their ID and FG number.

**Table 2:** Focus group analysis themes (Stage 1)

Theme	Sub-theme	Meaning	Illustrating citation
Determinants of PPI in numerical aspects of research	Relationship with researcher and research environment	Quality of interaction with research, including empathy and trust that facilitates and motivates patient and public partners	“**It’s doing it in such a way that you’re not devaluing the other person; you’re not making them feel as though they are stupid, and you really want to engage with them and understand their perspective, and that’s people skills.” [P2, FG1]**
	Patient and public partners confidence	Being able to question researchers and their assumptions	“**But I’d like to learn a bit more, so I could ask questions and start to be more effective in this kind of setting where there’s a whole bunch of things I don’t know about, as well as the numerical stuff. But I sense that the clinicians aren’t that confident either and they just get in a statistician as soon as they get to the numbers bit.” [P3, FG2]**
	Communication of numerical aspects	The use of jargon and the inaccessibility of definitions and resources to help patient and public partners participate in number related discussions	**“... You need wordsmiths. And there are very good technical writers who can write plain language, ... … You want the kind of science journalist that you see writing on the BBC website. Or contributing to radio and television programmes. You want someone that’s got a bit of scientific knowledge, you want someone that’s got some understanding of medical world...” [P1, FG2]**
	General perceptions of statistics and numbers	Observations about general public’s perceptions of statistics and numbers as a potential determinant of interest and understanding of research’s numerical aspects	**“… That’s quite scary when education’s so poor. Well it’s not the education that’s so poor, it’s the emphasis I think perhaps on numbers, there’s an awful lot of people very scared of numbers.” [P2, FG1]**
Identity and role	Patient and public partner’s role	What patient and public partners believe their role should be in relation to numerical aspects; their interest in helping define the context and assumptions behind deriving a number, as well as its interpretation	“**Yeah, it’s our job to decide what numbers you’re going to look for and then decide whether you’ve found the right numbers from our perspective and pass on that information.” [P5, FG1]**
	Patient and public partner’s characteristics	How motivation and personal experience can lead to more interest in being involved in numerical aspects of research; reflection on what that means for involvement in these aspects	“**For me the problem lies in when you ask these people to put a number, what influences them to reach the number five? There are the variables again you know, you’ve got your people who are poor, your people who are rich, your people who have false teeth and just put, “I’m not bothered”” [P5, FG1]**
Impact	Transparency	Ability to scrutinise researcher’s decisions on numerical aspects leads to more transparency in the whole process	“**I think transparency would be a main thing, like how can researchers, how can organisations, how can care providers be more transparent and disclose information which allows the patient, the public, to be able to make informed decisions.” [P4, FG2]**
	Feeling useful	Belief that numerical aspects are crucial in the research and policy making process and, therefore, being involved in them leads to a better understanding of the pathway and higher quality involvement	**“I think if I’m getting to improve or getting better at writing lay summaries, then I actually have to be able to understand the numerical aspects of the initial document that I have to read through, and then produce something which is then understandable without taking away from the meaning of the stats. [It is] about around understanding that journey, that pathway, and if you just provide the data without facilitating the understanding, then you just have numbers, you’re just dishing out numbers to someone and then they don’t understand.” [P4, FG2]**
	Improved research	As outsiders bringing questions in, patient and public partners can help improve the quality of the research done and disseminated	**“It’s the forcing them to think outside of their micro analytical numbers based box and forcing them to actually step into somebody else’s shoes and look at the work is actually intensely important however it’s done.” [P1, FG1]**
	Time consuming	Involvement is time consuming and this leads to exclusion of certain groups of people which is problematic; involvement in numerical aspects of research may be challenging due to taking time both for researchers and patient and public partners	“**I think for the researcher, it might be if you have got too many people questioning your research, and then that might make the process a bit longer, and then you’ve got all these deadlines that are coming up with the ethics panel, and all of these findings of patients.**“ **[P4, FG2]**

#### Theme 1: Determinants of patient and public involvement in numerical aspects of research

Theme 1 focused on factors identified by participants as influencing involvement in numerical aspects. These determinants were split into four sub-themes: relationship with researcher, self-confidence, communication of numerical aspects and their perception of the general public’s understanding and relationship with numbers and statistics.

Participants identified the research team’s environment and their relationship with researchers as key in making them feel comfortable with being involved in numerical aspects. Feeling safe and like you can speak up, having enough time to discuss topics, feeling the researchers have a flexible approach and listen to patient and public partners were all considered important facilitators.

“I think that is where a good researcher is worth their weight in gold you know, if you can engage with someone from the off and empathise with where they’re sitting at that moment in time and be interested, you can get a lot out of them.” [P2, FG1]

Numerical aspects were seen as particularly challenging to discuss and even intimidating for some participants, so the feeling of confidence in asking questions was highlighted as a determinant in whether patients and the public get involved. This was influenced by whether the environment felt safe and there was trust in the relationship with the researcher.

“So I think it’s having the right environment, it’s like you say, it’s getting the right people involved but not… it depends what you mean by the right people of course, but also having people that are maybe confident but not over confident and willing to ask the stupid question.” [P3, FG1]

Participants strongly emphasised the importance of communicating about numerical aspects in a clear and non-technical way to get patient and public partners involved. Lack of definitions of technical language available outside of meetings was a barrier in involving patients and the public with participants recommending alternative resources and modes of delivery of information to overcome this barrier.

“Somehow or another, I think there needs to be a statistics for dummies book. We had, several years ago now […] a speaker at one of the NCRI consumer forum meetings […] he came and talked to us, and somehow or another, statistics became very human, and it needs someone like him, someone with his skills, to write statistics for dummies.” [P1, FG2]

Determinants of patient and public involvement in numerical aspects mentioned above were seen as a reflection of a wider cultural and societal context, including the general public’s perceptions of statistics and numbers. This was both a potential challenge to get people interested in numerical aspects, but also a reason to involve patient and public partners in discussions to help make the connection between numbers and people.

“Could I raise my usual point here that about 20% of the Scottish and probably the UK population really struggle with numbers and being on the very verge of innumeracy, so that’s an awful lot of people, so I suppose any message must get across to the general public using diagrams or hail and damnation on the other side because “Our side is right and these numbers prove that they’re right”.” [P4, FG1]

#### Theme 2: Identity and role

Participants described what they think their role should be in numerical aspects. They also pointed out that patient or public partners’ characteristics may play a role in their interest and motivation in getting involved and that might bias the responses obtained.

Participants saw negotiating expectations about their potential role in numerical aspects of research as an essential first step so they have the opportunity to be more involved, if they wish to.

“when I get invited to join a panel, I ask for expectations, we set expectations to where do we sit in the greater scheme of things; how does this impact or influence x, and y, z. Are we just here to be a tick box exercise, or are we here to help, especially if they are tied to strategic blah, blah, blah?” [P4, FG2]

Participants felt strongly about the importance of involving patients and the public in the assumptions that go into defining numerical aspects and the analysis in research (i.e. at the start of process), but they were not as convinced about their involvement in the actual analysis. They were also interested on the translation and dissemination of the outputs to a wider audience.

“I think we need the opportunity to be asking questions during the analysis stage. So that we’re seeing interim data, we’re seeing the ideas evolving as the analysis proceeds, we need to be able to look at that and ask questions. We don’t need to be involved in how you calculate number here or an equation there.” [P1, FG2]

“So I think we should leave the statisticians to do the real heavy duty stuff, but I think we need to be involved at both the start and the end of the process so that the ordinary person in the street can understand and see what’s going on.” [P4, FG1]

Patient and public involvement can bring sense-checking and an outside perspective to numerical aspects.

“So in effect you need us because we’re actually pointing out to you, you’re trying to do what [name redacted] says you shouldn’t do, you’re trying to answer two questions at the same time [by asking about non-inferiority margins directly] and you’re bringing in variables and muddying the water so to speak.“ [P5, FG1]

When presented with the diagram showing the stages of the research lifecycle as well as the non-inferiority margin example, there was general agreement that patient and public partners should have the opportunity to discuss and contribute to the definition of target differences. However, there were disagreements about how (involvement in defining a number or in its assumptions?). Participants did not suggest any other numerical aspects to be involved in, except one (clinical equipoise). This seemed, at least partially, because patient and public partners felt uninformed about the wider research process and design of clinical trials.

Participants saw patient and public partner’s characteristics (their previous experience with numbers, their skills and their motivations) as key in whether they would have an interest in involvement in numerical aspects of trials and in how the communication of numerical aspects should be done.

“In one sense you might almost say you need a set of professional PPI people who kind of are self-aware enough to know that they shouldn’t push their agenda, have been to enough meetings so that they know when to shut up and when to just put their opinion across. But you’re always going to have problems with recruiting people off the street so to speak because you just don’t know what they’re bringing with them.” [P5, FG1]

#### Theme 3: Impact of involving patients and the public in numerical aspects

Theme 3 focuses on the potential impact that involving patients and the public can have. Participants felt being able to scrutinise researcher’s assumptions, including in the numerical aspects of research, and to discuss them openly would lead to more transparency in research; this was considered an extremely important and positive consequence of patient and public involvement and it can lead to better research.

“[…] so then it comes back to transparency, is there willingness from researchers, or from clinicians, or from the gatekeepers, whoever holds this information, for it to be accessible to patients. And I think if patients are involved in the start, then they can start to sort of highlight these issues or how is this going to be accessible to people afterwards.” [P4, FG2]

Another potential positive impact of involving patients and the public in numerical aspects is their feeling of accomplishment and of being able to provide more meaningful comments to lay summaries or discussions of results.

“Well, I think you get the opportunity to make a more meaningful contribution into the discussion [if patients and the public are involved in numerical aspects]. You know, there’s a lot of malarky about what impacts do involve patients have. Well, I have long believed that that’s the wrong way of looking at it, because what we do is we create a culture change and you don’t measure a culture change through simple impact in numbers” [P1, FG2]

In general, offering the opportunity of involving patients and the public in numerical aspects of research was seen as positive, but a potential issue was how time consuming this could be for both researchers and patient and public partners.

### Stage 2: Priority setting

The twelve numerical aspects identified in advance of the priority setting meeting are presented in Table 3. Clinical equipoise, suggested in the patient and public partner’s focus groups, had already been identified in our review. The priority setting meeting consisted of 14 stakeholders, comprising 3 patient partners, 1 chief investigator (clinical), 1 commissioner, 1 health economist, 1 trial methodologist (statistics), 1 trial coordinator, 1 trial manager, 1 patient and public involvement coordinator, 1 statistician, 2 experts in patient and public involvement in numerical aspects, 1 statistician/health economist.

**Table 3:** Numerical aspects selected to be discussed at the priority setting meeting (stage 2)

Aspects	Meaning
Target differences (clinically meaningful difference, non-inferiority margins)	This is the difference that will make researchers and clinicians conclude a treatment is better or good enough compared with a control
Risk/benefit trade-off	In a clinical trial, we usually test to find out whether a treatment gives more benefit than another. However, there could be risks or burdens to the patient there are different depending on the treatment.
Expected contamination	People in the control group unintentionally take the treatment or people in the treatment group unintentionally do not.
Clinical equipoise	A state of uncertainty in terms of what treatment option is best.
Randomisation allocation ratio	Ratio in which patients are allocated to receive a treatment compared with a control. It is usually done on a 1:1 basis which means the same number of people will get randomised to the treatment and the control.
Discussions about representativeness of sample	Discussion about the characteristics of people included in research studies and whether they are representative of the population of interest
Recruitment and retention projections	Recruitment is the process through which an individual is recruited as a study participant. Participant retention is the engagement of the participant in the research study.
Stop/go criteria	Often trials include specific criteria to decide on whether they should move forward, i.e. collect all data as planned or stop due to unfeasibility of recruitment, treatment delivery or due to treatment harm.
Data monitoring committee data discussions	A committee that may be established by the trial sponsor to assess at intervals, the progress of a clinical trial, the safety data, and the critical efficacy endpoints, and to recommend to the sponsor whether to continue, modify, or stop a trial. (INVOLVE; webpage consulted in 09/11/2020)
Missing data	When a participant outcome is unavailable, due to a missing questionnaire or non-attendance to a trial related clinical appointment
Cost-effectiveness (value for money)	Economic analysis that views effects in terms of overall health specific to the problem, and describes the costs for some additional health gain (e.g. cost per additional stroke prevented).
Interpretation of trial results and their dissemination	Discussion about trial results (presented as numbers, for example, treatment effects) and how to present them to patients and the public

**Fig. 2:**
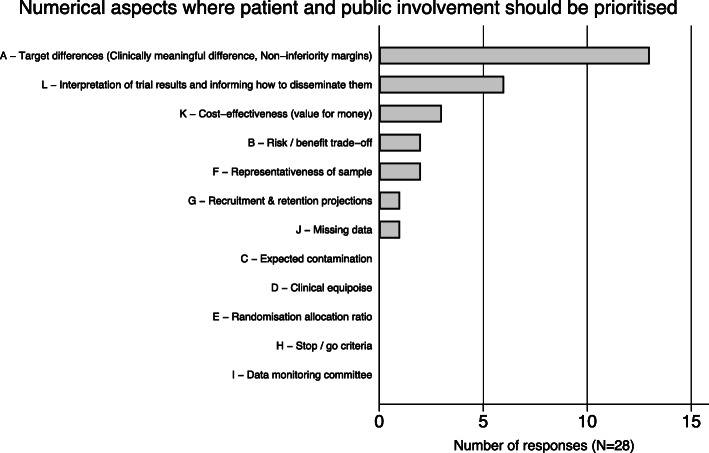
Number of responses that classified each numerical aspect as their top 2; each participant could vote twice so the total is 28 (14 participants × 2) (stage 2)

Figure 2 shows the final ordering of the numerical aspects prioritised. The top three aspects selected were target differences, interpretation of results and cost-effectiveness. Expected contamination, clinical equipoise, randomisation allocation, stop/go criteria and data monitoring committees were not part of any stakeholders’ top 2.

During the discussions that supported the voting process, participants felt that involvement in numerical aspects of trials was extremely important and that prioritisation could help achieve it, but all numerical aspects should be considered for future involvement and research. There was discussion on the weight of responsibility of selecting three priority aspects on behalf of a wider group of stakeholders and that led to nervousness in some participants. Participants felt that any discussion about numerical aspects needs to start with common ground definitions about what these aspects are to ensure a meaningful discussion and avoid power imbalances. Feelings of anxiety when discussing numerical aspects were expressed due to their technical nature. After the voting in the final plenary session, there was a discussion about the top 3. All participants felt that the final top 3 reflected their small group discussions throughout the morning. Target differences were consistently selected as part of participant’s top 3 because they were seen as the closest numerical aspects to patient’s experience (“what is meaningful to patients?”); interpretation of results and dissemination of findings were considered important to improve communication with patients and trial participants and to ensure implementation of findings; value for money was also seen as close to patient’s experience and as a key piece in health policy decision making.

## Discussion

We have conducted the first study exploring patient and public partners’ experiences and motivations regarding their involvement in numerical aspects of research and trials, as well as the first priority setting exercise identifying what numerical aspects of trials are the most important for patients and the public to contribute to. We found that patients and the public believe their involvement in numerical aspects is important, particularly in its assumptions and communication, and could lead to improved transparency and more meaningful involvement. Stakeholders selected target differences, interpretation of results and cost-effectiveness as their top 3 numerical aspects of trials to involve patients and the public in.

Communication of and jargon related to numerical aspects was identified as a key barrier to patient and public involvement. This is not new—Gamble et al. raised this issue almost ten years ago [16], but it has clearly remained a challenge [17]. Other determinants of patient and public involvement in numerical aspects of research raised were the environment and relationship with researchers which has been found to be a main influencer on patient and public involvement quality [18]. This can affect self-confidence which can, in turn, affect patient and public partners’ perceived ability to question researchers. This is in line with focus group findings discussing patient and public involvement in trials [17], and the determinants found here are also the foundation to building strong and productive working relationships between researchers and patient or public partners, the number one methodological priority for PPI in trials [7]. General perceptions about numbers and statistics, as well as numeracy levels, may influence whether people decide to get involved in numerical aspects of research; they may also be the reason why patient and public involvement is important to these aspects—a way of translating numbers into stories. Any strategy to achieve this needs to account for the low levels of numerical and statistical literacy in the general population [8] and adapt communication to reach a diverse group of people.

Patients and the public perceived their role in numerical aspects as sense-checkers bringing an outside perspective supporting past self-descriptions of PPI contributors as “challenging outsiders” [19]. It was suggested their involvement should happen in a funnel format: helping researchers develop the assumptions that go into defining numbers and statistical models and aiding interpretation and dissemination of results. Their interest in the “statistical nitty-gritty” was less obvious, as they felt like they did not have the expertise to be involved. This proposed model of involvement goes in line with Hannigan’s reflections of PPI in statistics [8]: involving patients and the public in the actual analysis process might be an inefficient use of PPI resources since it demands considerable technical knowledge; however, quantitative data are numbers with a context and patient and public partners could help statisticians identify and understand that context. We found patient and public partner’s role expectations should be set up from the start which agrees with other research about patient and public involvement in general [5]. A lot of the patient partners felt like they did not have enough information about stages of research or numerical aspects of trials and recommended resources such as induction books and presentations as potential solutions.

Who can and would like to be involved in numerical aspects of trials was raised in the focus groups, with the perception that personal experience may affect motivation and that patients might be biased when giving their opinions on numerical aspects. This issue is raised in research related to PPI in health economics modelling with suggestions that patients involved need to be able to keep a neutral view [9]. In previous work, we found around half of UK trialists responding a survey about PPI believed patient and public partners had to have specific skills to be involved in numerical aspects; these included numeracy, statistical knowledge and understanding of trials. However, other fields outside of health research have used participatory methods to involve non-experts in model development [20] and other statistical aspects [21].

Target differences (clinically meaningful differences and non-inferiority margins) were selected by 13 out of the 14 stakeholders as one of their top 2 numerical aspects to involve patients and the public. Methods to include patient’s views on clinically meaningful differences indirectly exist, via for example anchor-based approaches. However, the relevance of their methods and results is not currently discussed with patients [22]. When it comes to direct elicitation or opinion seeking methods for clinically meaningful differences, patients have been involved but their focus tends to be on clinicians [23]. Researchers have not, to our knowledge, involved patients and the public in defining non-inferiority margins despite recent calls to do so [24] and controversies over how current margins are defined [25].

Interpretation and discussion of results was selected by 6 out of 14 stakeholders as one of their top 2 numerical aspects. The importance of appropriate dissemination of trial results and patient and public involvement in its development has only recently been on the research agenda [26] and, even though there is some evidence on how to communicate numerical evidence to the public [27], its role in trials needs to be further explored. Cost-effectiveness was selected by 3 out of 14 stakeholders as their top 2 numerical aspect. Methods to involve patient and public in cost-effectiveness and health economics in trials have been recently discussed [28] with experts believing patients are a key stakeholder in this process [9].

## Strengths and limitations

Our study was initially planned to be delivered face-to-face (focus groups and priority setting meeting) and had to be adapted to an online setting given the COVID-19 pandemic. Online settings might have advantages (such as wider geographical reach), but they also have disadvantages (exclusion of potential participants that do not have access to the technology needed; lack of non-verbal cues may lead to more difficult communication). However, the facilitators pro-actively attempted to ensure that the views of all the participants were heard—checking back with each individual to ensure all their points had been covered. We also advertised the focus groups widely and ensured it was clear we would provide support for participants to take part if needed. We worked to adapt our methods and followed James Lind Alliance guidance on the best practice to conduct online priority setting meetings. Focus group participants and stakeholders that accepted to take part in our priority setting meeting are, however, likely to have a particular interest in the topic and might not represent the views of other patient and public partners or experts in trials. Moreover, patient and public partners may have higher education and possibly numeracy levels than the general population [19]. Numerical aspects of trials are challenging to discuss due to their technical nature and, for that reason, we provided a thorough guide of what we meant by each aspect, as well as a video presentation of what the priority setting meeting would entail—this is a strength of the study. We also explicitly addressed nervousness and the potential for power imbalances from the start of the meeting, reassuring everyone about their important voice in the conversation, and splitting up the groups to allow each patient/public partner to stay with the same facilitator throughout. Going forward, we would recommend a pre-meeting to discuss any queries related to the technical aspects as well as the opportunity for patient partners to meet up in a separate online room to discuss their views. Patient and public partners, as well as the other stakeholders, felt like their views were considered and reflected on the top three priorities.

## Conclusion

Many trial key decisions are based on numbers. Keeping numbers out of the discussion with patients, and/or making the discussion difficult by not communicating about them appropriately, can perpetuate power imbalances between patient and public partners and researchers [19]. In order to ensure PPI in trials is not tokenistic, patients should be given the opportunity to contribute to its numerical aspects. We have shown that for patient and public views to be appropriately heard, the environment in which these discussions are held is important (allowing ample time for discussion; where researchers listen; and where any perceived lack of confidence with numbers is supported); jargon needs to be omitted and lay definitions provided, and the relationship and trust established between the research team and the patients and public is paramount. Our research provides a platform for future efforts to improve patient and public involvement in trials and a prioritised set of future research foci.

## Future research recommendations

Future research should focus on improving involvement of patients and the public in numerical aspects of trials. This will unavoidably involve developing methods to improve communication about statistics and numerical aspects, including training materials that allow more meaningful conversations between patient partners and researchers. The training materials could be aimed at both patient partners (to improve their understanding of the impact of statistics in trials and clinical practice) and trialists (to improve communication about numerical aspects). Materials aiming to explain numerical aspects should be developed to reach people independent of their numerary levels. There is scope to explore the adaptation of methods such as Bayesian elicitation [29], typically used with clinicians, to incorporate patient’s views in numerical aspects of trials. These types of methods could be used to discuss all numerical aspects presented in our work, but their acceptability and feasibility need to be assessed before implementation in trials.

## Data Availability

Data are available upon reasonable request.
